# Supplementary material for: Assessing Different Chronic Wasting Disease Training Aids for Use with Detection Dogs
Source: Animals (Basel). 2024 Jan 18;14(2):300. doi: 10.3390/ani14020300 (PMC10812555; doi:10.3390/ani14020300)
Supplement: Supplementary file 1 [file animals-14-00300-s001.zip › animals-2768959-supplementary.pdf]

Supplementary Tables
Table S1: Training and Testing Phases

Stage	Target Odor	Negative and Distractor Odors Used	Description	Criteria	Time in Stage –Trained Dogs	Time in Stage – New Dogs
Pre-Training	UDC*	Distractors: laboratory odors (alcohol, paper towel, etc)	Learning search mechanics	Dogs can search an 8-port scent wheel, find odor, perform trained final alert at odor, and perform a "blank behavior" when there is no odor present	Variable; two dogs began as puppies, and the other four began as participants in our community science program	
CWD Imprinting	Fecal matter from CWD+ deer	Negative: Fecal matter from CWD- deer	New dogs learned CWD+ fecal matter odor. The positive sample is shown to the dog and they are rewarded for sniffing it. They then are asked to find this sample in a lineup.	Dogs can find fecal matter from CWD+ deer in 80% of trials in one session after they are rewarded for sniffing the positive sample	N/A	4-6 sessions
CWD Training	Fecal matter from CWD+ deer	Negative: Fecal matter from CWD- deer<br><br>Distractors: laboratory odors (alcohol, paper towel, etc) and unimpregnated training aids	Dogs found positive samples in a five-port lineup in preparation for the training aid test stage.	Dogs can find the positive sample in a lineup at 80% sensitivity and 80% specificity on initial encounter with odor	5-8 sessions	13-19 sessions
Training Aid Tests	Training Aids incubated with fecal matter from CWD+ deer	Negative: Training aids incubated with fecal matter from CWD- deer<br><br>Distractors: unimpregnated and control training aids	Dogs were tested on novel training aids. Each trial either contained a positive, negative, and control of the same type of training aid (e.g., cotton aid incubated for 24 hours at 21 C) or was a blank, containing only distractors and controls.	Labrador Retriever	4 test sessions, 6-7 trials per session (depending on number of blanks), for a total of 27 test trials. Because Charlie began having mechanical difficulties searching, he did 18 trials (no blanks) and free-searched rather than searching in order.	4 test sessions, 6-7 trials per session (depending on number of blanks), for a total of 27 test trials

*Universal Detector Calibrant, a training odor

Table S2

CWD ethogram used to code dogs' behavior in the five-port lineup.

Behavior	Type (Duration or Point)	Description
Session duration	Duration	Session begins when dog first enters the room and ends when the dog has finished the last trial of the session
Trial	Duration	Trial begins when dog begins searching the lineup and ends when the dog either 1) correctly alerts or performs blank behavior and is clicked for the behavior; or 2) incorrectly alerts or incorrectly calls a blank and is called out from the search area
Duration at port	Duration	Starts when dog's nose is within 6 inches of port, ends when dog is further than 6 inches from port
Sit at port	Point	Dog sits in front of port
